# Integrating International Consensus Guidelines for Anticancer Drug Dosing in Kidney Dysfunction (ADDIKD) into everyday practice

**DOI:** 10.1016/j.eclinm.2025.103161

**Published:** 2025-03-25

**Authors:** Geeta Sandhu, Josephine Adattini, Evangeline Armstrong Gordon, Niamh O'Neill, Corrinne Bagnis, Alan V. Boddy, Pinkie Chambers, Alex Flynn, Brett Hamilton, Karim Ibrahim, David W. Johnson, Christos Karapetis, Aisling Kelly, Kimberley-Ann Kerr, Ganessan Kichenadasse, David S. Kliman, Craig Kurkard, Winston Liauw, Catherine Lucas, Andrew J. Mallett, Jolanta Malyszko, Georgia McCaughan, Michael Michael, Sanja Mirkov, Emma Morris, Carol A. Pollock, Darren M. Roberts, David J. Routledge, Carla Scuderi, Julia Shingleton, Jake Shortt, Jim Siderov, Ben Sprangers, Brian N. Stein, Amanda Tey, Kate Webber, Jenny Wichart, Rachel Wong, Robyn L. Ward, ADDIKD Working Group

**Affiliations:** a Faculty of Medicine and Health, The University of Sydney, Sydney, NSW, Australia; b eviQ, Cancer Institute NSW, St Leonards, NSW, Australia; c Pharmacy Department, St Vincent's Hospital, Sydney, NSW, Australia; d Nephrology Department, APHP Sorbonne University, and GRIFON, Paris, France; e School of Clinical and Health Sciences, University of South Australia, Adelaide, SA, Australia; f University College London School of Pharmacy and University College London Hospital-University College London Centre for Medicines Optimisation Research and Education, London, United Kingdom; g Centre for Drug Repurposing, University of Newcastle, Newcastle, NSW, Australia; h Albury Wodonga Cancer Care, West Albury, NSW, Australia; i Northeast Health Wangaratta, Wangaratta, VIC, Australia; j Albury Wodonga Health, Albury, NSW, Australia; k Faculty of Medicine and Health, The University of New South Wales, Sydney, NSW, Australia; l Australasian Kidney Trials Network, The University of Queensland, Brisbane, QLD, Australia; m Department of Kidney and Transplant Services, Princess Alexandra Hospital, Brisbane, QLD, Australia; n Translational Research Institute, Brisbane, QLD, Australia; o Flinders Centre for Innovation in Cancer, Flinders Medical Centre/Flinders University, Bedford Park, SA, Australia; p SA Health, Adelaide, SA, Australia; q Department of Haematology, Royal North Shore Hospital, Sydney, NSW, Australia; r University of Newcastle, Newcastle, NSW, Australia; s Cancer Care Centre, St George Hospital, Kogarah, NSW, Australia; t School of Clinical Medicine, The University of New South Wales, Sydney, NSW, Australia; u Department of Renal Medicine, Townsville University Hospital, Townsville, QLD, Australia; v College of Medicine & Dentistry, James Cook University, Townsville, QLD, Australia; w Institute for Molecular Bioscience, The University of Queensland, Brisbane, QLD, Australia; x Department of Nephrology, Dialysis and Internal Medicine, Warsaw Medical University, Warsaw, Poland; y Department of Haematology, St Vincent's Hospital, Sydney, NSW, Australia; z Garvan Institute of Medical Research, Sydney, NSW, Australia; aa Peter MacCallum Cancer Centre, Parkville, VIC, Australia; ab The Sir Peter MacCallum Department of Oncology, The University of Melbourne, Melbourne, VIC, Australia; ac Pharmacy Department, Cairns and Hinterland Hospital and Health Service, Cairns, QLD, Australia; ad School of Pharmacy, University of Queensland, Brisbane, QLD, Australia; ae Pharmacy Department, University College London Hospitals NHS Foundation Trust, London, United Kingdom; af Kolling Institute Medical Research, Sydney, NSW, Australia; ag Edith Collins Centre, Drug Health Services, Royal Prince Alfred Hospital, Camperdown, NSW, Australia; ah Clinical Haematology, Peter MacCallum Centre and Royal Melbourne Hospital, Parkville, VIC, Australia; ai Pharmacy Department, Royal Brisbane and Women's Hospital, Brisbane, QLD, Australia; aj Monash Haematology, Monash Health, Clayton, VIC, Australia; ak Department of Medicine, School of Clinical Sciences at Monash Health, Monash University, Clayton, VIC, Australia; al Pharmacy Department, Austin Health, Melbourne, VIC, Australia; am Biomedical Research Institute, Department of Immunology and Infection, UHasselt, Diepenbeek, Belgium; an Department of Nephrology, Ziekenhuis Oost Limburg, Genk, Belgium; ao ICON Cancer Centre, Adelaide, SA, Australia; ap The University of Adelaide, Adelaide, SA, Australia; aq Pharmacy Department, Monash Health, Clayton, VIC, Australia; ar Department of Medical Oncology, Monash Health, Clayton, VIC, Australia; as Alberta Health Services, Alberta, Canada; at Department of Medical Oncology, Eastern Health, Box Hill, VIC, Australia; au Eastern Health Clinical School, Monash University, Box Hill, VIC, Australia

**Keywords:** Chemotherapy, Drug dosing, Kidney dysfunction, Renal, Oncology, Haematology, Pharmacokinetics

## Abstract

Part 2 of the International Consensus Guideline on Anticancer Drug Dosing in Kidney Dysfunction (ADDIKD) offers drug-specific consensus recommendations based on both evidence and practical experience. These recommendations build upon the kidney function assessment and classification guidelines established in Part 1 of ADDIKD. Here we illustrate how dosing recommendations differ between ADDIKD and existing guidance for four commonly used drugs: methotrexate, cisplatin, carboplatin and nivolumab. We then describe how the recommendations can be distilled into practice points for methotrexate and cisplatin. While ADDIKD is a significant improvement from previous guidelines, adoption of this new guideline requires further endorsement from key external stakeholders, ‘change championing’ by clinicians locally and encouraging its integration into existing reference sources, clinical trial protocols and electronic prescribing systems.

**Funding:**

Development of the ADDIKD guideline is funded by the 10.13039/501100021708NSW Government as part of the 10.13039/501100001171Cancer Institute NSW and received no funding from external commercial sources.

**Research in context**

**Evidence before the study**

High quality evidence for anticancer drug dosing in reduced kidney function is limited and no internationally agreed guidelines exist to inform prescribing decisions in this population.

**Added value of this study**

The International Guideline for Anticancer Drug Dosing in Kidney Dysfunction (ADDIKD) standardised the assessment of kidney function (Part 1) and its application to anticancer drug dosing using published evidence and expert consensus (Part 2). Here, we have selected four widely prescribed anticancer drugs (methotrexate, cisplatin, carboplatin and nivolumab) to illustrate how ADDIKD's drug specific recommendations compare to previously published guidance. The challenges of implementing ADDIKD into clinical practice are also discussed.

**Implications of all the available evidence**

An internationally standardised, evidenced- and consensus-based approach to the dosing cancer patients with abnormal kidney function was much needed resource clinically, and adoption into regulatory drug processes within government and the pharmaceutical industry envisaged. Ongoing review of emerging evidence, inclusion of new anticancer drugs and incorporation of patients on kidney replacement therapy into ADDIKD will need to be considered for future updates.

## Introduction

The International Consensus Guideline for Anticancer Drug Dosing in Kidney Dysfunction (ADDIKD)^1^ was developed in two parts, with Part 1 providing three recommendations for the assessment and classification of kidney function in cancer patients [unpublished].^2^ In Part 2, consensus recommendations for 59 anticancer drugs were formulated on both evidence and practice-based decisions [unpublished]^3^ (see Supplementary Material 1 for a summary of ADDIKD's drug specific recommendations).

ADDIKD has been widely endorsed internationally, including by the International Society of Geriatric Oncology, British Oncology Pharmacy Association, Haematology Society of Australia and New Zealand, Medical Oncology Group of Australia, Clinical Oncology Society of Australia, Australasian Society of Clinical and Experimental Pharmacologists and Toxicologists, Advanced Pharmacy Australia [AdPha] (formerly Society of Hospital Pharmacists of Australia), Australian and New Zealand Urogenital and Prostate Cancer Trials Group and The UK Renal Pharmacy Group. Kidney Disease: Improving Global Outcomes (KDIGO), AdPha and the American Society of Onco-Nephrology have included ADDIKD's recommendations in their position statements and guidance.

In this paper, we have selected four widely prescribed anticancer drugs (methotrexate, cisplatin, carboplatin and nivolumab) to illustrate how ADDIKD's drug specific recommendations compare to previously published guidance such as the Renal Drug Database,^4^ regulatory-approved product information,^5^ dose adjustment recommendations by BC Cancer's drug monographs,^6^ articles by Krens and colleagues,^7^^,^^8^ and Australia's eviQ treatment protocols (pre-ADDIKD implementation into its protocols [before July 2023]).^9^ For methotrexate and cisplatin, we demonstrate how ADDIKD can be practically incorporated into a dosing guide for cancer clinicians.

## Exemplar anticancer drugs

### Methotrexate

Methotrexate is an anticancer drug administered via several routes (orally and parenterally) and used in the management of multiple malignancies including those requiring high plasma concentrations to achieve adequate tumour cell kill.^10^^,^^11^ Methotrexate has a wide dosing range and is associated with high variability in interpatient pharmacokinetics.^12^ The adverse event profile depends on the dosing (and consequential drug exposure), and high doses (≥ 500 mg/m^2^) can cause serious adverse events including acute kidney injury (AKI).^13^^,^^14^

Existing guidelines propose methotrexate dose adjustments in reduced kidney function (Table 1); however, there are notable inconsistencies such as the kidney function threshold for discontinuation (varies between creatinine clearance [CrCl] or glomerular filtration rate [GFR] of 10–30 mL/min). Furthermore, there is little information on how tumour type, intent of treatment or dosing levels should influence dose recommendations.

**Table 1:** Comparison of ADDIKD's recommendations vs existing guidance for methotrexate dosing according to kidney function.

Abbreviations: CrCl, creatinine clearance calculated using the Cockcroft–Gault equation; eGFR, estimated glomerular filtration rate via the Chronic Kidney Disease—Epidemiology Collaboration equation; GFR, glomerular filtration rate (not standarised to body surface area); KRT, kidney replacement therapy.

a In patients with curative intent, good performance status and no concomitant nephrotoxic drugs.

b Certain protocols with higher doses of methotrexate had more conservative dose adjustments.

As shown in Table 1, ADDIKD's guidance distinguishes high-from low-dose methotrexate and curative from non-curative treatment intent (see Supplementary Material 2 for ADDIKD's complete methotrexate dosing recommendations).^1^ Unlike other guidance, when prescribing high-dose methotrexate, ADDIKD strongly recommends the utilisation of directly measured glomerular filtration rate (through direct measurement of the clearance of exogenous markers such as iohexol, iothalamate, ^51^Cr-EDTA or ^99m^Tc-DTPA and expressed in mL/min)^15^^,^^16^ at baseline rather than an estimated assessment (i.e., estimated glomerular filtration rate [eGFR], CrCl). By way of a specific example, overall survival of patients with primary central nervous system lymphoma (PCNSL) relies on high-dose methotrexate to penetrate the blood–brain-barrier.^17^^,^^18^ Existing guidelines empirically halve the dose of methotrexate when CrCl or GFR is 20–50 mL/min, whereas ADDIKD enables tailoring of the extent of dose reduction, by considering treatment intent and/or additional patient factors into more discrete bands according to the KDIGO Chronic Kidney Disease (CKD) categories of kidney function. Being curative, high-dose methotrexate in PCNSL patients (with a good performance status) who have an eGFR between 45–59 mL/min/1.73 m^2^ (or directly measured GFR between 45–59 mL/min) should maintain full doses, thereby ensuring dose exposure without significantly increasing toxicity.^19^^,^^20^ ADDIKD recommends a 25% dose reduction or using an alternative protocol in patients not receiving curative treatment and/or poorer performance status. For example, if considering the use of low-dose methotrexate protocols, such as for breast cancer, alternative regimens without methotrexate demonstrate similar survival outcomes and do not increase the risk haematological and kidney-related adverse events.^21^, ^22^, ^23^

**Fig. 1:**
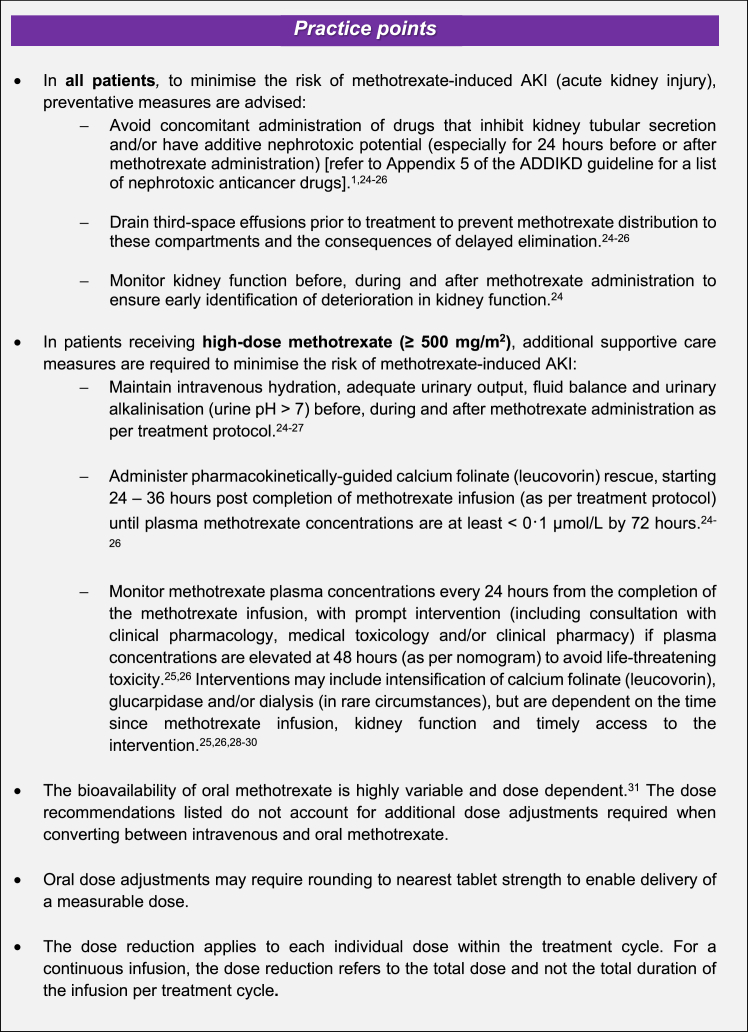
Methotrexate dosing practice points from ADDIKD.^1^

In addition to the specific dosing adjustments shown in Table 1, ADDIKD includes a set of ‘practice points’ to guide the administration of methotrexate (see Fig. 1).

### Cisplatin

Cisplatin can be nephrotoxic,^24^^,^^25^ with 20–50% excreted by the kidneys.^26^, ^27^, ^28^ In addition, it has broad dosing ranges and is used in the treatment of many different tumours. Despite the widespread use of cisplatin, there is a paucity of evidence supporting dose adjustment recommendations in patients with reduced kidney function. Several studies have reported significantly poorer overall survival in patients with eGFR < 60 mL/min/1.73 m^2^ who received reduced doses compared to patients with normal kidney function receiving full doses (at ≥ 50 mg/m^2^ [inclusive of total fractionated doses]).^29^, ^30^, ^31^

In the absence of robust evidence, existing guidelines suggest generalised dose adjustments for a range of tumour types without tailoring to treatment intent or dosing ranges (Table 2). Existing guidelines do not wholly consider factors which increase kidney-related adverse events such as concomitant nephrotoxic drug usage,^32^^,^^33^ and performance status of the patient.^32^ Large variations in dose adjustments for reduced kidney function are evident, with some guidelines recommending 25–50% dose reduction for CrCl or GFR 30–59 mL/min whilst others suggest when CrCl or GFR is < 40 mL/min, cisplatin is contraindicated.

**Table 2:** Comparison of ADDIKD's recommendations vs existing guidance for cisplatin dosing according to kidney function.

Abbreviations: CrCl, creatinine clearance calculated using the Cockcroft–Gault equation; eGFR, estimated glomerular filtration rate via the Chronic Kidney Disease—Epidemiology Collaboration equation; GFR, glomerular filtration rate (not standarised to body surface area); KRT, kidney replacement therapy.

a In patients with curative intent, good performance status and no concomitant nephrotoxic drugs.

b In patients with either a poor performance status or concomitant nephrotoxic drugs.

ADDIKD's guideline for cisplatin incorporates several new factors for dose consideration. Firstly, dividing cisplatin dosing into high versus low-dose with a cut-off dosing level of 50 mg/m^2^ [inclusive of total fractionated doses] (Table 2).^1^ This dosing determination was based on the potential risk of high peaks of free platinum concentrations leading to cisplatin-induced adverse kidney events. High peaks are associated with doses > 50 mg/m^2^, more frequent administration, a larger cumulative dose, and hypoalbuminaemia.^34^, ^35^, ^36^, ^37^ Secondly, dose adjustment recommendations were aligned with KDIGO CKD categories of kidney function and therefore tailor dose adjustments to more discrete bands compared to existing guidance.

Particularly in the eGFR 45–59 mL/min/1.73 m^2^ category, ADDIKD recommends a dose reduction of high-dose cisplatin or considering an appropriate alternative treatment protocol with specific consideration of performance status and/or concomitant nephrotoxic drug exposure (see Supplementary Material 3 for ADDIKD's complete cisplatin dosing recommendations) [refer to Appendix 5 of the ADDIKD guideline for a list of nephrotoxic anticancer drugs].^1^ In the supporting text of the guideline, ADDIKD gives the option of splitting full dose cisplatin into divided doses a week apart, thereby enabling patients with a good performance status with certain cancers (such as advanced urothelial cancer) to receive gold-standard treatment.^38^^,^^39^ This is preferable to omitting or dose reducing cisplatin as per current guidelines.

ADDIKD also tailors its guidance for patients treated with curative intent. Patients with a good performance status receiving ≤ 50 mg/m^2^ of cisplatin in the absence of concomitant nephrotoxic drugs are recommended full dose at eGFR 45–59 mL/min/1.73 m^2^. Lelli et al., showed the rates of vomiting, haematological toxicities, and kidney-related adverse events in patients with reduced kidney function (eGFR 40–59 mL/min/1.73 m^2^) receiving dose adjusted cisplatin (40–70% dose reduced) were comparable to those with normal kidney function receiving full dose cisplatin (50 mg/m^2^).^40^ In line with current guidelines, ADDIKD recommended avoiding cisplatin when eGFR < 45 mL/min/1.73 m^2^, given there is no significant evidence to support its safe use in reduced kidney function, especially when alternative, less nephrotoxic treatment regimens may be appropriate.

**Fig. 2:**
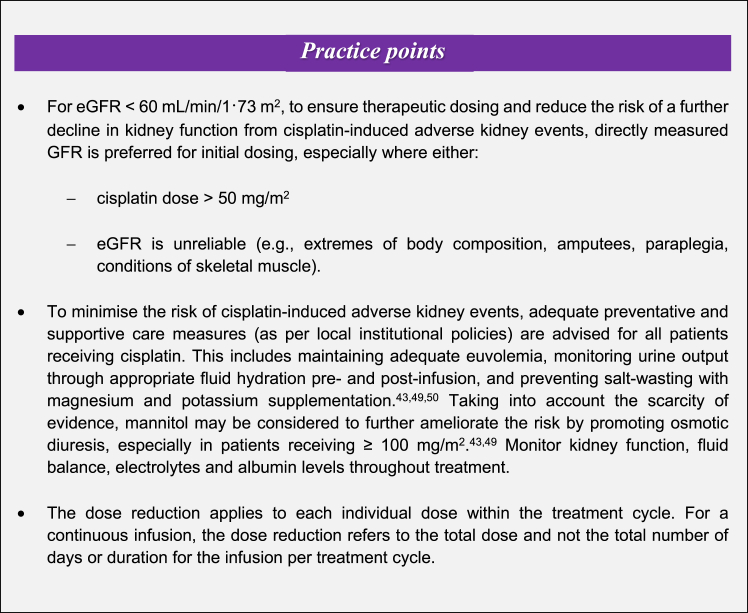
Cisplatin dosing practice points from ADDIKD.^1^

Finally, in contrast to existing guidelines, ADDIKD recommends preventative and supportive care measures, and advocates for the use of directly measured GFR prior to the administration of high-dose cisplatin or where eGFR is unreliable (see Fig. 2 for ‘practice points’ for cisplatin). The latter includes patients with extremes of body composition, amputees, paraplegia, or conditions of skeletal muscle.^1^ Measuring GFR in these circumstances prevents escalation of kidney-related adverse events in patients with reduced kidney function. Estimated kidney function values may underestimate true kidney function leading to unnecessary omission of cisplatin as a treatment option.

### Carboplatin

Carboplatin is used in both oncological and haematological malignancies, with its dose calculation being directly reliant on actual kidney function. This is because carboplatin clearance is linearly proportional to kidney function, with elimination by the kidneys largely dependent on glomerular filtration rate and only a minor reliance on tubular secretion.^41^^,^^42^ A strong correlation exists between carboplatin area under the curve (AUC), kidney function and the severity of thrombocytopenia, and, to a lesser extent, leucopoenia,^43^, ^44^, ^45^, ^46^, ^47^, ^48^ Therefore in contemporary practice the Calvert formula uses kidney function and AUC to calculate carboplatin doses, leading to less grade ≥ 3 myelosuppression (based on National Cancer Institute Common Terminology Criteria for Adverse Events [CTCAE])^49^ whilst maintaining therapeutic efficacy in patients with eGFR < 60 mL/min/1.73 m^2^.^44^^,^^50^

Besides regulatory-approved product information, existing guidelines recommend the Calvert formula (Table 3). ADDIKD did not recommend KDIGO CKD categories in guiding the dosing of carboplatin in reduced kidney function, but rather the use of the Calvert formula (see Supplementary Material 4 for ADDIKD's complete carboplatin dosing recommendations).^1^

**Table 3:** Comparison of ADDIKD's recommendations vs existing guidance for carboplatin dosing according to kidney function.

Abbreviations: AUC, area under the concentration–time curve; BSA-adjusted eGFR, body surface area adjusted estimated glomerular filtration rate via the Chronic Kidney Disease—Epidemiology Collaboration equation (mL/min); CrCl, creatinine clearance calculated using the Cockcroft–Gault equation; eGFR, estimated glomerular filtration rate via the Chronic Kidney Disease—Epidemiology Collaboration equation; MDT, multidisciplinary team consisting of oncology/haematology with nephrology and/or clinical pharmacology for the management of dosing; mGFR, directly measured glomerular filtration rate (not standarised to body surface area, mL/min); KRT, kidney replacement therapy.

a When either treatment intent is curative or patient has extremes of body composition, conditions of skeletal muscle, is an amputee or is paraplegic or eGFR > 125 mL/min/1.73 m^2^ or eGFR ≤ 45 mL/min/1.73 m^2^

There is inconsistency amongst existing guidelines given the heterogenous use of kidney function assessments applied to the Calvert formula, potentially creating significant variation in calculated doses.^51^ ADDIKD proposes that directly measured GFR (not standardised to body surface area [BSA]) as the preferred kidney function value to be used to mirror the original Calvert formula study.^44^ This ensures that with curative intent, or in clinical situations where estimated kidney function is unreliable (including eGFR > 125 mL/min/1.73 m^2^ or ≤ 45 mL/min/1.73 m^2^), directly measured GFR provides for more consistent therapeutic dosing.

The ADDIKD guideline acknowledges that access to and affordability of directly measured GFR can be unfeasible in some settings. In these situations where the decision is made to use estimated kidney function values instead of the gold standard directly measured GFR, the use of BSA-adjusted estimated GFR as the kidney function value in the Calvert formula is recommended by expert clinical consensus. AUC calculated using eGFR (via the Chronic Kidney Disease—Epidemiology Collaboration [CKD-EPI] equation), when adjusted for an individual's BSA (calculated through either DuBois DuBois or Mosteller equations)^52^^,^^53^ in the Calvert formula, is more accurate than AUC calculated using CrCl via the Cockcroft–Gault equation.^54^, ^55^, ^56^, ^57^ Since ADDIKD's publication, KDIGO's Clinical Practice Guideline for the Evaluation and Management of CKD,^58^ Advanced Pharmacy Australia,^59^ and the American Society of Onco-Nephrology^60^ have supported the use of BSA-adjusted eGFR for carboplatin dose calculations over CrCl (when directly measured GFR is not feasible).

The change to BSA-adjusted eGFR was identified as a significant practice change, based on historical reliance on CrCl calculated by the Cockcroft–Gault equation. Furthermore, many internationally used oncology prescribing software packages employ CrCl as the default kidney function value in the Calvert formula.

To support networks yet to transition to electronic prescribing software and align with ADDIKD recommendations, eviQ (Cancer Institute NSW's web-based government program for point of care information for health professionals in Australia [www.eviq.org]) developed a ‘rapid learning module’ as an online educational learning tool for carboplatin dose calculation.^9^ An online calculator for carboplatin dosing based on both directly measured GFR and BSA-adjusted eGFR was also developed.

In contrast to existing guidelines, ADDIKD incorporates additional practice-based recommendations to standardise approaches to calculation of carboplatin doses. This includes a recommendation against lowering target AUC in reduced kidney function, as it may compromise clinical benefit. The recalculation of carboplatin doses at each cycle was also deemed unnecessary, except when baseline kidney function (e.g., eGFR) alters by > 20% or when there is a change in the clinical status of the patient.^1^

### Nivolumab

As a monoclonal antibody, nivolumab is not pharmacokinetically reliant on kidney function for drug elimination,^61^, ^62^, ^63^ and has a low incidence of CTCAE grade ≥ 3 or treatment-limiting toxicities in patients with reduced kidney function.^64^, ^65^, ^66^, ^67^, ^68^, ^69^, ^70^, ^71^ Nivolumab illustrates that although an anticancer drug may appear safe at any level of kidney function, precautions still exist that should be considered on an individual patient level. As a newer anticancer immunotherapy agent, evidence of nivolumab tolerability in eGFR < 30 mL/min/1.73 m^2^ is only now emerging. Immune-related adverse kidney events have been observed with nivolumab treatment, and commonly involve AKI, arising from acute interstitial nephritis, acute tubular injury, or glomerular diseases.^68^^,^^70^^,^^72^, ^73^, ^74^, ^75^, ^76^, ^77^

The impact of reduced kidney function at baseline on the risk of immune-related adverse kidney events with nivolumab is uncertain, with some studies reporting no association^72^^,^^74^^,^^76^^,^^77^ and another observing an increased risk of immune-checkpoint inhibitor-associated AKI with declining kidney function.^78^ ADDIKD notes the developing toxicity data, especially as nivolumab use expands amongst wider patient populations, as well as in combination with nephrotoxic agents [refer to Appendix 5 of the ADDIKD guideline for a list of nephrotoxic anticancer drugs].^1^

Existing guidance does not recommend dose adjustments to nivolumab in the presence of reduced kidney function.^5^, ^6^, ^7^, ^8^, ^9^ ADDIKD similarly does not suggest dose adjustments, however provides additional considerations for high-risk situations (e.g., kidney transplant recipients, patient groups susceptible to developing immune-related AKI) and monitoring for immune-related kidney events (see Supplementary Material 5 for ADDIKD's complete nivolumab dosing recommendations).^1^

In agreement with several international guidelines,^79^^,^^80^ ADDIKD recommends assessment of baseline kidney function, and measurement of electrolyte levels and urinalysis before starting and as clinically indicated throughout nivolumab treatment to monitor for immune-related kidney events. This is pertinent in patients with additional risk factors for developing immune-related AKI (such as concomitant nephrotoxic drug exposure, combination immune checkpoint inhibitor therapy, dehydration, and pre-existing hypertension).^72^^,^^74^^,^^76^, ^77^, ^78^

#### The next steps for implementation of ADDIKD into clinical practice

These drug exemplars demonstrate how clinical decision-making for dosing patients with reduced kidney function can be standardised whilst accommodating individual patient and treatment-related factors. Compared to existing guidance, ADDIKD integrates evidence with clinical expertise to formulate a justified pragmatic dosing recommendation for individual drugs in the presence of reduced kidney function. It also provides a traffic-light colour-coded, easy to read, ‘quick reference’ dosing tables summarising guidance for multidisciplinary cancer teams (including members who are less familiar with anticancer drugs or treatment protocols). Although only 59 drugs were evaluated in ADDIKD, the methodology of assessment could be utilised by clinicians for other anticancer drugs in the future. Additionally, ADDIKD's integration into cancer treatment resources (i.e., eviQ, BC Cancer) and clinical trial protocols would aid in the global standardisation of anticancer drug dose adjustment in reduced kidney function.

Local ‘change champions’, from multiple disciplines (i.e., oncologists/haematologists, nephrologists, pharmacists, nurses), are imperative in leading the implementation of ADDIKD's principles—primarily using eGFR for estimated assessment, harmonising the categorisation of kidney function to KDIGO and application of the relevant dosing recommendations. We recommend that cancer practices employ a multidisciplinary team review of current policies and workflow processes. This could include an assessment of electronic prescribing system capabilities including the use of auto-calculation of carboplatin doses using the Calvert formula. Likewise, identifying which of their patients would benefit from a directly measured GFR assessment and determining local alternatives for kidney function assessment in such patients if directly measured GFR is impractical.

Local pathology service providers should be consulted to ensure eGFR is reported using the CKD-EPI equation. Additionally, directly measured GFR values should be reported appropriately as mL/min and not as a BSA standardised value.

Depending on local capacity, the adoption of ADDIKD in cancer centres should occur in a staged manner to allow for gradual familiarisation. One approach could include incorporating ADDIKD dosing recommendations for all drugs except for carboplatin. After a period of familiarisation, the cancer centre may wish to consider the major change of adopting BSA-adjusted eGFR for carboplatin prescribing (where directly measured GFR was not feasible). Ultimately, a ‘lead by example’ approach from institutional ‘change champions’ is required along with clear advocacy for the advantages ADDIKD can bring to the practice, building on the implementation of several major ADDIKD principles, completing pre and post implementation audits and recruiting the other members of the team to further develop dose optimising changes.

#### Outstanding questions

There are several ADDIKD limitations to consider during implementation and for future updates to the guideline. Firstly, stem cell mobilisation, bone marrow transplantation, cellular therapies and kidney replacement therapies were outside the scope of the guidance. These are highly complex areas with limited data and would benefit from a consensus guideline process as comprehensive as ADDIKD. Secondly, inclusion of newer drugs over time and updating existing guidance as new evidence emerges. The process of consensus building, and guideline development has been established, however the requirement for guideline continuity and updates in this rapidly evolving area in medicine is difficult without definitive resource commitment. The initial publication was through a government funded project and involved clinicians volunteering their expertise. Thirdly, inclusion of newer and more precise methods of kidney function estimation. For example, recently the utilisation of cystatin C-creatinine measured estimation equations have been shown to be more accurate in clinical situations where eGFR is unreliable.^58^

## Contributors

Conceptualisation—GS, RLW, JShi, AK, CS, BNS, WL, NO.

Data curation—GS, RLW, JA, NO, DMR, AJM, PC.

Formal analysis—GS, RLW, JA, NO, DMR, EM, AVB, BNS, AJM, MM, JSho.

Investigation—GS, RLW, EAG, JA, CK, AJM, KW, MM, DK, DJR, JSho, KW.

Methodology—GS, RLW, DMR, DWJ, CS, MM, BNS, DJR, NO.

Project administration—GS, RWL, NO, JA, AK, JShi, CS, MM.

Resources—GS, RLW, BNS, MM.

Supervision—GS, RLW, JShi, AK, MM.

Validation—GS, RLW, EAG, JA, AJM, DMR, KW, MM, BS.

Visualisation—GS, RLW, JA, EAG, NO, MM, PC.

Writing—original draft—GS, RLW.

Writing—reviewing/editing–all authors.

All authors provided critical revision of the manuscript for important intellectual content and gave approval of the submission of the manuscript for publication. GS, RLW and JA accessed and verified data in the study for publication, and GS and RLW had final responsibility for the decision to submit for publication.

## Data sharing statement

None.

## Declaration of interests

AJM reports a relationship with National Health and Medical Research Council that includes: funding grants. AJM reports a relationship with Australian Government Medical Research Future Fund that includes: funding grants. AJM reports a relationship with Queensland Government that includes: funding grants. AJM reports a relationship with Townsville Hospital and Health Service that includes: funding grants. AJM reports a relationship with Otsuka Pharmaceutical Co Ltd that includes: travel reimbursement. AJM reports a relationship with GSK that includes: travel reimbursement. AJM reports a relationship with Australia and New Zealand Society of Nephrology that includes: board membership. JS reports a relationship with Roche that includes: speaking and lecture fees. JS reports a relationship with Amgen Inc that includes: speaking and lecture fees. JS reports a relationship with AbbVie Inc that includes: speaking and lecture fees. JS a relationship with Pfizer Inc that includes: speaking and lecture fees. JS reports a relationship with Mundipharma International Limited that includes: speaking and lecture fees. JS reports a relationship with Bristol Myers Squibb Co that includes: speaking and lecture fees. JS reports a relationship with Baxter Health that includes: speaking and lecture fees. JS reports a relationship with Becton Dickinson and Company that includes: speaking and lecture fees. JS reports a relationship with Juniper Pharmaceuticals Inc that includes: board membership. JS reports a relationship with Antengene Corporation Limited that includes: board membership. DWJ reports a relationship with Baxter Health that includes: speaking and lecture fees. JSho reports a relationship with Astex Pharmaceuticals US Corporate and Development Headquarters that includes: funding grants. JSho reports a relationship with Astellas Pharma Inc that includes: consulting or advisory. JSho reports a relationship with Otsuka Pharmaceutical Co Ltd that includes: consulting or advisory. JSho reports a relationship with Novartis Pharmaceuticals Corporation that includes: consulting or advisory and speaking and lecture fees. JSho reports a relationship with Pfizer Inc that includes: consulting or advisory. JSho reports a relationship with Mundipharma International Limited that includes: speaking and lecture fees. JSho reports a relationship with Bristol Myers Squibb Co that includes: board membership. JSho reports a relationship with Victoria Cancer Council that includes: board membership. JSho reports a relationship with Australian Leukaemia and Lymphoma Group that includes: board membership. GM reports a relationship with Janssen that includes: speaking and lecture fees, travel reimbursement. GM reports a relationship with Pfizer that includes: speaking and lecture fees. BS reports a relationship with Omeros Corporation that includes: consulting or advisory. BS reports a relationship with AstraZeneca Pharmaceuticals LP that includes: speaking and lecture fees and travel reimbursement. BS reports a relationship with Vifor Pharma Switzerland SA that includes: speaking and lecture fees. BS reports a relationship with Janssen Pharmaceuticals Inc that includes: speaking and lecture fees. BS reports a relationship with Boehringer Ingelheim Corp USA that includes: board membership and speaking and lecture fees. BS reports a relationship with MorphoSys US Inc that includes: board membership. GK reports a relationship with Cancer Council SA that includes: board membership. SM reports a relationship with AdPha that includes: board membership. EM reports a relationship with Novartis that includes: speaking and lecture fees. EM reports a relationship with Lilly that includes: travel reimbursement. PC reports a relationship with Pfizer that includes: funding grants. PC reports a relationship with Gilead Sciences Inc that includes: funding grants, speaking and lecture fees, and travel reimbursement. RLW reports a relationship with NSW Cancer Institute that includes: board membership. RLW reports a relationship with the Pharmaceutical Benefits Advisory Committee, Commonwealth of Australia that includes: board membership. If there are other authors, they declare that they have no known competing financial interests or personal relationships that could have appeared to influence the work reported in this paper.
