# Supplementary material for: Integrating International Consensus Guidelines for Anticancer Drug Dosing in Kidney Dysfunction (ADDIKD) into everyday practice
Source: eClinicalMedicine. 2025 Mar 25;82:103161. doi: 10.1016/j.eclinm.2025.103161 (PMC12034076; doi:10.1016/j.eclinm.2025.103161)
Supplement: Supplementary Material [file mmc1.docx]

**Supplementary Appendix to: Integrating International Consensus Guidelines for Anticancer Drug Dosing in Kidney Dysfunction (ADDIKD) into everyday practice**

Supplementary Material 1……………………………………Page 2

Supplementary Material 2……………………………………Page 4

Supplementary Material 3……………………………………Page 6

Supplementary Material 4……………………………………Page 8

Supplementary Material 5…………………………………..Page 10

References………………...…………………………………Page 11

**Supplementary Material 1**

**Summary of dosing recommendations for 59 individual anticancer drugs according to KDIGO CKD categories for kidney function.^1^**

| **eGFR**  **(mL/min/1.73 m^2^)** | **Dose** ^a^ | **Drugs** |
| --- | --- | --- |
| 45 – 59 | **full dose** | Recommended for   - cabazitaxel, cetuximab, chlorambucil, dabrafenib, docetaxel, doxorubicin, epirubicin, nivolumab, paclitaxel, panitumumab, pembrolizumab, thalidomide, vinblastine, vincristine, vindesine, vinorelbine.   Suggested for   - azacitidine, bendamustine, bortezomib, cyclophosphamide, low-dose cytarabine (< 1000 mg/m^2^), dacarbazine, *pegylated liposomal* doxorubicin, durvalumab, idarubicin, oxaliplatin, nab-paclitaxel, pertuzumab, procarbazine, rituximab, temozolomide, trastuzumab, trastuzumab emtansine. |
|  | **full dose** | Recommended, but kidney function may inform the monitoring of adverse events for   - bleomycin, etoposide (including etoposide *phosphate*), obinutuzumab, venetoclax.   Suggested, but kidney function may inform the monitoring of adverse events for   - bevacizumab, dactinomycin, daunorubicin (including *liposomal* daunorubicin), everolimus, gemcitabine, ifosfamide, irinotecan, mitomycin, pemetrexed, thiotepa. |
|  | **dose reduction**  **and/or**  **alternative protocol** | Recommended for   - capecitabine, cisplatin, fludarabine, lenalidomide, methotrexate, raltitrexed, topotecan.   Suggested for   - high-dose cytarabine (≥ 1000 mg/m^2^), fluorouracil, melphalan, mercaptopurine, vinflunine. |
|  | **target AUC using Calvert formula** | Suggested, but kidney function may inform the monitoring of adverse events for   - carboplatin. |
| 30 – 44 | **full dose** | Recommended for   - cabazitaxel, cetuximab, dabrafenib, docetaxel, doxorubicin, epirubicin, nivolumab, paclitaxel, panitumumab, pembrolizumab, thalidomide, vinblastine, vincristine, vindesine, vinorelbine.   Suggested for   - azacitidine, bendamustine, bortezomib, cyclophosphamide, low-dose cytarabine (< 1000 mg/m^2^), dacarbazine, *pegylated liposomal* doxorubicin, durvalumab, idarubicin, oxaliplatin, nab-paclitaxel, pertuzumab, rituximab, temozolomide, trastuzumab, trastuzumab emtansine. |
|  | **full dose** | Recommended, but kidney function may inform the monitoring of adverse events for   - chlorambucil, obinutuzumab, venetoclax.   Suggested, but kidney function may inform the monitoring of adverse events for   - bevacizumab, dactinomycin, daunorubicin (including *liposomal* daunorubicin), everolimus, gemcitabine, irinotecan, mitomycin, thiotepa. |
|  | **dose reduction**  **and/or**  **alternative protocol** | Recommended for   - bleomycin, capecitabine, etoposide (including etoposide *phosphate*), fludarabine, lenalidomide, methotrexate, raltitrexed, topotecan.   Suggested for   - high-dose cytarabine (≥ 1000 mg/m^2^), fluorouracil, ifosfamide, melphalan, mercaptopurine, pemetrexed, procarbazine vinflunine. |
|  | **target AUC using Calvert formula** | Suggested, but kidney function may inform the monitoring of adverse events for   - carboplatin. |
|  | **AVOID** | Recommended for   - cisplatin. |
| 15 – 29 | **full dose** | Recommended for   - cabazitaxel, cetuximab, dabrafenib, docetaxel, doxorubicin, epirubicin, nivolumab, paclitaxel, panitumumab, pembrolizumab, vinblastine, vincristine, vindesine, vinorelbine.   Suggested for   - low-dose cytarabine (< 1000 mg/m^2^), durvalumab, pertuzumab, rituximab, trastuzumab, trastuzumab emtansine. |
|  | **full dose** | Recommended, but kidney function may inform the monitoring of adverse events for   - chlorambucil, thalidomide.   Suggested, but kidney function may inform the monitoring of adverse events for   - azacitidine, bendamustine, bevacizumab, bortezomib, dactinomycin, *pegylated liposomal* doxorubicin, everolimus, gemcitabine, nab-paclitaxel, temozolomide, thiotepa. |
|  | **dose reduction**  **and/or**  **alternative protocol** | Recommended for   - bleomycin, etoposide (including etoposide *phosphate*), lenalidomide, obinutuzumab, venetoclax.   Suggested for   - cyclophosphamide, dacarbazine, daunorubicin (including *liposomal* daunorubicin), fluorouracil, idarubicin, ifosfamide, irinotecan, melphalan, mercaptopurine, oxaliplatin, procarbazine, vinflunine. |
|  | **target AUC using Calvert formula** | Suggested, but kidney function may inform the monitoring of adverse events for   - carboplatin. |
|  | **AVOID** | Recommended for   - capecitabine, cisplatin, fludarabine, methotrexate, raltitrexed, topotecan.   Suggested for   - high-dose cytarabine (≥ 1000 mg/m^2^), mitomycin, pemetrexed. |
| < 15    (without  KRT) | **full dose** | Recommended for   - cetuximab, docetaxel, doxorubicin, nivolumab, panitumumab, pembrolizumab, vinblastine, vincristine, vindesine, vinorelbine.   Suggested, for   - low-dose cytarabine (< 1000 mg/m^2^), durvalumab, pertuzumab, rituximab, trastuzumab, trastuzumab emtansine. |
|  | **full dose** | Recommended, but kidney function may inform the monitoring of adverse events for   - cabazitaxel, paclitaxel, thalidomide.   Suggested, but kidney function may inform the monitoring of adverse events for   - bendamustine, bevacizumab, bortezomib. |
|  | **dose reduction**  **and/or**  **alternative protocol** | Recommended for   - lenalidomide, venetoclax. |
|  | **AVOID** | Recommended for   - bleomycin, capecitabine, cisplatin, fludarabine, methotrexate, raltitrexed, topotecan.   Suggested for   - high-dose cytarabine (≥ 1000 mg/m^2^), mitomycin, pemetrexed. |
|  | Consult a multidisciplinary team consisting of oncology/haematology with nephrology and/or clinical pharmacology for the management of dosing. | Recommend for   - chlorambucil, dabrafenib, epirubicin, etoposide (including etoposide *phosphate*), obinutuzumab.   Suggested for   - azacitidine, carboplatin, cyclophosphamide, dacarbazine, dactinomycin, daunorubicin (including *liposomal* daunorubicin), *pegylated liposomal* doxorubicin, everolimus, fluorouracil, gemcitabine, idarubicin, ifosfamide, irinotecan, melphalan, mercaptopurine, oxaliplatin, nab-paclitaxel, procarbazine, temozolomide, thiotepa, vinflunine. |
| KRT | Consult a multidisciplinary team consisting of oncology/haematology with nephrology and/or clinical pharmacology for the management of dosing. | |
| **^a^** Proposed dosing under specific circumstances | | |
| *Abbreviations: eGFR, estimated glomerular filtration rate via the Chronic Kidney Disease – Epidemiology Collaboration equation; KRT, kidney replacement therapy.* | | |

**Supplementary Material 2**

**ADDIKD’s dose recommendations for methotrexate.^1^**

| **ORAL and INTRAVENOUS METHOTREXATE DOSE RECOMMENDATIONS** ^a^ | | | | |
| --- | --- | --- | --- | --- |
| **eGFR**  **(mL/min/1.73 m^2^)** | **Dose** | | **Comment** | |
|  |  |  |  |  |
| ≥ 60 | **full dose** ^b,c,d^ | |  |  |
| 45 – 59 | **When protocol *starting dose* is**  **< 500 mg/m^2^**  **alternative protocol**  **or**  **reduce by 25%** ^b,c,e,f,g^ | **When protocol *starting dose* is**  ≥ **500 mg/m^2^**  **full dose** ^b,c,d^    **or**  **reduce by**  **25%** ^b,c.d,f^  **or**  **alternative protocol** | In **< 500 mg/m^2^**, consider a clinically appropriate alternative treatment protocol in patients with:   - curativ*e* treatment intent, *and* - poor performance status, *and* - concomitant nephrotoxic drug exposure.   In all other patients, consider a 25% dose reduction.  In **≥ 500 mg/m^2^,** consider full dose in patients with:   - curative treatment intent, where maintaining an exposure threshold is required (i.e., primary CNS lymphoma), *and* - good performance status, *and* - no concomitant nephrotoxic drug exposure   In all other patients, consider a 25% dose reduction or a clinically appropriate alternative treatment protocol.  Increased risk of adverse events (i.e., haematological toxicities [myelosuppression], gastrointestinal toxicities [mucositis], AKI). |  |
| 30 – 44 | **alternative protocol**  **or**  **reduce by 50%** ^b,c,d,e,f,g^ | | Consider a clinically appropriate alternative treatment protocol in patients with a curative treatment intent or in patients with:   - poor performance status, *and* - concomitant nephrotoxic drug exposure   In all other patients, consider a 50% dose reduction.  Increased risk of adverse events (i.e., haematological toxicities [myelosuppression], gastrointestinal toxicities [mucositis], AKI). |  |
| 15 – 29 | **AVOID** | | Not recommended – use a clinically appropriate alternative treatment protocol. | |
| < 15   (without KRT) |  |  |  |  |
| KRT | Consult a multidisciplinary team consisting of oncology/haematology with nephrology and/or clinical pharmacology for the management of dosing. | | | |
| ^a^ For bone marrow transplantation protocols involving graft versus host disease prophylaxis, consult the transplant team if the patient has kidney dysfunction and is requiring methotrexate as part of their treatment. The dose adjustments have not been tailored for these protocols.  ^b^ To ensure therapeutic dosing and reduce the incidence of methotrexate-related adverse events, directly measured GFR is preferred for the initial dosing especially where *either*:   - eGFR is < 60 mL/min/1.73 m^2^ - Methotrexate doses are ≥ 500 mg/m^2^ - eGFR may be unreliable in specific clinical circumstances (e.g., extremes of body composition, amputees, paraplegia, conditions of skeletal muscle).   Measured GFR refers to a direct measurement of the clearance of exogenous markers such as iohexol, iothalamate, 51Cr-EDTA (radioactive chromium complex with ethylenediaminetetraacetic acid) or ^99^Tc-DTPA (TC-diethylenetriaminepentaacetic acid).  ^c^ The following preventative measures are advised to minimise methotrexate-induced AKI in all patients:   - Avoid concomitant use of drugs that impair kidney elimination of methotrexate or have additive nephrotoxic potential (especially 24 hours either side of methotrexate doses) - Drain third space effusions prior to treatment - Monitor kidney function before, during and after methotrexate administration.   ^d^ For doses ≥ 500 mg/m^2^ additional supportive care measures are required to minimise methotrexate-indued AKI:   - Maintain intravenous hydration, adequate urinary output, fluid balance and urinary alkalinisation (pH > 7) before, during and after methotrexate administration as per treatment protocol. - Use pharmacokinetically-guided calcium folinate (leucovorin) rescue starting 24-36 hours post methotrexate infusion (as per treatment protocol) until plasma methotrexate concentrations are at least < 0.1 µmol/L by 72 hours. - Monitor methotrexate plasma concentrations every 24 hours from the end of the methotrexate infusion, with prompt intervention if plasma concentrations are high at 48 hours (as per nomogram) to avoid life-threatening toxicity.   ^e^ The bioavailability of oral methotrexate is highly variable and dose dependent. The dose recommendations listed do not account for additional dose adjustments required when converting between intravenous and oral methotrexate.  ^f^ The dose reduction applies to each individual dose within the treatment cycle. For a continuous infusion, the dose reduction refers to the total dose and not the total duration of the infusion per treatment cycle**.**  ^g^ Dose adjustments may require rounding to nearest tablet strength to enable delivery of a measurable dose. | | | | |
| *Abbreviations: AKI – acute kidney injury; eGFR, estimated glomerular filtration rate via the Chronic Kidney Disease – Epidemiology Collaboration equation; KRT, kidney replacement therapy.* | | | | |

**Supplementary Material 3**

**ADDIKD’s dose recommendations for cisplatin.^1^**

| **INTRAVENOUS CISPLATIN DOSE RECOMMENDATIONS** | | | |
| --- | --- | --- | --- |
| **eGFR**  **(mL/min/1.73 m^2^)** | **Dose** | | **Comment** |
| ≥ 60 | **full dose** ^a^ | |  |
| 45 – 59 | **When protocol starting dose is**  **≤ 50 mg/m^2^**  **full dose** ^a,b^  **or**  **reduce by 25%** ^a,b,c^  **or**  **alternative protocol** | **When protocol starting dose is**  **> 50 mg/m^2^**  **alternative protocol** ^d^  **or**  **reduce by 25 – 50%** ^a,b,c^ | In **> 50 mg/m^2^** (inclusive of total fractionated doses),  consider a clinically appropriate alternative treatment protocol especially in patients with *either*:   - a poor performance status - concomitant nephrotoxic drug exposure.   In all other patients, if proceeding with cisplatin, consider a 25 – 50% dose reduction. Extent of dose reduction should take into account:   - intent of treatment - performance status - potential total cumulative cisplatin exposure.   In **≤ 50 mg/m^2^** (inclusive of total fractionated doses), consider full dose in patients with:   - curative treatment intent, *and* - a good performance status, *and* - without concomitant nephrotoxic drug exposure.   In all other patients, consider a 25% dose reduction or a clinically appropriate alternative treatment protocol.  Potential for increased risk of adverse events (i.e., kidney-related toxicities [especially when risk factors present]^e^**,** haematological toxicities, nausea and vomiting) |
| 30 – 44 | **AVOID** | | Not recommended – use a clinically appropriate alternative treatment protocol. |
| 15 – 29 |  |  |  |
| < 15   (without KRT) |  |  |  |
| KRT | Consult a multidisciplinary team consisting of oncology/haematology with nephrology and/or clinical pharmacology for the management of dosing. | | |
| ^a^ Adequate preventative and supportive care measures (as per local institutional policies) are advised for all patients to minimise the risk of cisplatin-induced kidney adverse events and include:   - Intravenous hydration, magnesium, and potassium supplementation +/- mannitol - Monitoring kidney function, urine output, electrolytes, albumin, and fluid balance throughout treatment.   ^b^ To ensure therapeutic dosing and reduce the risk of a further decline in kidney function from cisplatin-induced adverse kidney events, directly measured GFR is preferred for the initial dosing especially where *either* cisplatin dose > 50 mg/m^2^ or eGFR is unreliable (e.g., extremes of body composition, amputees, paraplegia, conditions of skeletal muscle). Measured GFR refers to a direct measurement of the clearance of exogenous markers such as iohexol, iothalamate, ^51^Cr-EDTA (radioactive chromium complex with ethylenediaminetetraacetic*)* or ^99^Tc-DTPA (TC-diethylenetriaminepentaacetic acid).  ^c^ The dose reduction applies to each individual dose within the treatment cycle. For a continuous infusion, the dose reduction refers to the total dose and not the total number of days or duration for the infusion per treatment cycle.  ^d^ Clinically appropriate alternative treatment protocols for selected patients in certain cancers may include protocols that split cisplatin doses a week apart.  ^e^ Risk factors for developing cisplatin-induced adverse kidney events include high peaks of free platinum concentrations (possibly caused by doses > 50 mg/m^2^, more frequent administration, larger cumulative dose, and hypoalbuminaemia), hypertension, concomitant nephrotoxic drug exposure, older age, and poor performance status. | | | |
| *Abbreviations: eGFR, estimated glomerular filtration rate via the Chronic Kidney Disease – Epidemiology Collaboration equation; KRT, kidney replacement therapy.* | | | |

**Supplementary Material 4**

**ADDIKD’s dose recommendations for carboplatin.^1^**

| **INTRAVENOUS CARBOPLATIN DOSING RECOMMENDATION** ^a^ | | |
| --- | --- | --- |
| **eGFR**  **(mL/min/1.73 m^2^)** | **Dose** | **Comment** |
|  |  |  |
| ≥ 60 | **target AUC using Calvert formula** ^b,c^ | **Directly measured GFR**^d^ is the preferred kidney function value in the Calvert formula, especially when *either*:   - treatment intent is curative - patient has extremes of body composition, conditions of skeletal muscle, is an amputee or is paraplegic - eGFR > 125 mL/min/1.73 m^2^.   If estimating kidney function, **BSA-adjusted eGFR**^e^ is preferred as the kidney function value in the Calvert formula.  Capping of kidney function is not recommended^f^. |
| 45 – 59 | **target AUC using Calvert formula** ^b,c^ | **Directly measured GFR**^d^ is the preferred kidney function value in the Calvert formula especially when *either*:   - treatment intent is curative - patient has extremes of body composition, conditions of skeletal muscle, is an amputee or is paraplegic.   If estimating kidney function, **BSA-adjusted eGFR**^e^ is preferred as the kidney function value in the Calvert formula.  Increased risk of adverse events (i.e., thrombocytopenia, leucopenia) especially in patients with either a poor performance status, extensive prior anticancer treatment, or concomitant nephrotoxic drug exposure. |
| 30 – 44 | **target AUC using Calvert formula** ^b,c^ | **Directly measured GFR**^d^ is the preferred kidney function value in the Calvert formula.  Increased risk of adverse events (i.e., thrombocytopenia, leucopenia) especially in patients with either a poor performance status, extensive prior anticancer treatment, or concomitant nephrotoxic drug exposure. |
| 15 – 29 |  |  |
| < 15   (without KRT) | Consult a multidisciplinary team consisting of oncology/haematology with nephrology and/or clinical pharmacology for the management of dosing. | |
| KRT |  |  |
| ^a^ For bone marrow transplantation conditioning protocols, consult the transplant team if the patient has kidney dysfunction and is requiring carboplatin as part of their treatment. The dose adjustments have not been tailored for these protocols.  ^b^ Recalculation of carboplatin doses at each cycle is unnecessary, except when baseline kidney function (e.g., eGFR) alters by > 20% or when there is a change in the clinical status of the patient.  ^c^ Calvert formula: dose (mg) = target AUC (mg mL^-1^ min) × [GFR (mL/min) + 25 (mL/min)]  ^d^ Measured GFR refers to a direct measurement of the clearance of exogenous markers such as iohexol, iothalamate, ^51^Cr-EDTA (radioactive chromium complex with ethylenediaminetetraacetic acid) or ^99^Tc-DTPA (TC-diethylenetriaminepentaacetic acid).  ^e^ BSA-adjusted eGFR (mL/min) via the CKD-EPI equation= [eGFR (mL/min/1.73 m^2^) × BSA (m^2^)] ÷ 1.73. Use either Mosteller or DuBois DuBois equations to calculate BSA. Online calculator available at: <https://www.eviq.org.au/p/4171>  ^f^ Capping kidney function to 125 mL/min/1.73 m^2^ for use in the Calvert formula may reduce therapeutic efficacy without reducing toxicity. When automated laboratory eGFR values are reported as greater than an upper limit (e.g., eGFR ≥ 90 mL/min/1.73 m^2^), manual calculation of eGFR via the CKD-EPI equation is required before applying this value to the BSA-adjusted eGFR in the Calvert formula. | | |
| *Abbreviations: AUC, Area under the concentration-time curve; BSA, body surface area; CKD-EPI, Chronic Kidney Disease Epidemiology Collaboration; eGFR, estimated glomerular filtration rate via the CKD-EPI equation; KRT, kidney replacement therapy.* | | |

**Supplementary Material 5**

**ADDIKD’s dose recommendations for nivolumab.^1^**

| **INTRAVENOUS NIVOLUMAB DOSE RECOMMENDATIONS** | | |
| --- | --- | --- |
| **eGFR**  **(mL/min/1.73 m^2^)** | **Dose** | **Comment** |
| ≥ 60 | **full dose** ^a^ |  |
| 45 – 59 |  |  |
| 30 – 44 |  |  |
| 15 – 29 |  |  |
| < 15  (without KRT) |  |  |
| KRT | Consult a multidisciplinary team consisting of oncology/haematology with nephrology and/or clinical pharmacology for the management of dosing. | |
| ^a^ Measurement of baseline kidney function, electrolyte levels and urinalysis are advised before commencement and as clinically indicated throughout nivolumab treatment to monitor for developing immune-related kidney adverse events. This is particularly pertinent in patients with additional risk factors for developing immune-related AKI (i.e., concomitant nephrotoxic drug exposure, combination immune checkpoint inhibitor therapy, dehydration, pre-existing hypertension). | | |
| *Abbreviations: AKI, acute kidney injury; eGFR, estimated glomerular filtration rate via the Chronic Kidney Disease – Epidemiology Collaboration equation; KRT, kidney replacement therapy.* | | |

**References**

1. Sandhu G, Adattini J, Gordon EA, O’Neill N, On behalf of the ADDIKD Guideline Working Group. International consensus guideline on anticancer drug dosing in kidney dysfunction. 2022. <https://www.eviq.org.au/clinical-resources/addikd-guideline/4174-anticancer-drug-dosing-in-kidney-dysfunction> (accessed 31 July 2024).
